# Metacognition and Intersubjectivity: Reconsidering Their Relationship Following Advances From the Study of Persons With Psychosis

**DOI:** 10.3389/fpsyg.2020.00567

**Published:** 2020-03-25

**Authors:** Ilanit Hasson-Ohayon, Andrew Gumley, Hamish McLeod, Paul H. Lysaker

**Affiliations:** ^1^Department of Psychology, Bar-Ilan University, Ramat Gan, Israel; ^2^Institute of Health and Wellbeing, University of Glasgow, Glasgow, United Kingdom; ^3^Roudebush VA Medical Center, Indianapolis, IN, United States; ^4^School of Medicine, Indiana University–Purdue University Indianapolis, Indianapolis, IN, United States

**Keywords:** psychosis, metacognition, intersubjectivity, psychopathology, psychotherapy

## Abstract

As research on metacognition has progressed a significant array of definitions, methodologies and therapeutic applications have emerged. Some of this work has primarily framed metacognition as an activity carried out by one person in order to know, monitor, and adjust their beliefs, memories, and behaviors. Accordingly, problems with metacognition have often been characterized as issues related to cognition. This, however, risks neglecting how metacognition is also a fundamentally intersubjective act, one in which human beings know and reflect upon themselves and others primarily with and through connections with other people. In this paper, we review research on metacognition in schizophrenia using the integrative model of metacognition and a research paradigm in which metacognition is assessed within personal narratives. Stimulated by this work, we discuss how disturbances in intersubjective experience and metacognitive capacity mutually influence one another, with disruptions in metacognition perhaps more deeply understood as disruptions in relatedness with others. We then discuss how metacognition and intersubjectivity each affect mental health. We finally focus on the implications of this for treatments that target metacognition as well as future directions for research.

## Introduction

The term metacognition can be traced back to its introduction in a paper by Flavell (1979) on developments in education (Moritz and Lysaker, 2018). In this initial piece, Flavell integrated ideas from developmental and educational psychology (e.g., Vygotsky, 1997), and emphasized the value of thinking about thinking as people progress developmentally and acquire new skills and information. In this first paper, Flavell (1979) emphasized how metacognition involves the conscious awareness of cognitive performance and listed four components: metacognitive knowledge—stored beliefs about self and others; metacognitive experiences—reflection about cognitive processes; metacognitive goals; and metacognitive tasks. This broad definition has served as the groundwork for recent literature highlighting how a person’s appraisal of their own mental activities can deeply influence how effectively persons are able to respond to challenges to their health and wellness (Lysaker and Klion, 2017).

Metacognition, as foreshadowed by Flavell, has come to be regarded as a complex group of activities. These commonly include processes related to recognizing individual thoughts, emotions, and bodily states and reflecting on their relationship to events within the flow of daily life. Metacognition can involve recursive processes in which individuals’ specific experiences are interpreted on the basis of an awareness of the larger context in which those specific experiences occur. For example, failure at a task may be understood and attributed to one or more factors in the context of other failures and successes. Broader ideas about the patterns and qualities of individuals’ lives are then understood as evolving as these specific experiences are incorporated to related other experiences. For example, an appraisal of one’s competence may be naturally affected by how a series of specific successes or failures are understood (Moritz and Lysaker, 2018). Of note, to a large extent many contemporary approaches to metacognition, including Flavell’s relatively later work (Flavell et al., 2002), emphasize the practical purposes of metacognitive knowledge and the self-regulatory aspects of metacognition. These components are evident in Moritz and Lysaker (2018) theoretical discussion of metacognition and the Lysaker et al. (2018) review of different metacognitive approaches to the treatment of psychosis which treat metacognitive processes as having a regulatory function that allows person to doubt and subsequently decide upon different courses of action. One aspect of metacognition, however, that is often overlooked, is that metacognition is entwined with intersubjective experience. Instead of a cognitive activity concretely located in an isolated mind, metacognition involves phenomena which occur between persons. Appraisals of experiences, including one’s own thoughts, wishes, and feelings as well as the outcome of behavior, are necessarily shaped by the relative meanings assigned to different aspects of that experience. Those meanings that are assigned to an aspect of experience are naturally influenced and molded by how others do or might perceive and think about either those experiences or to how one is interpreting those experiences (Hasson-Ohayon et al., 2017). For example, thoughts about the effectiveness of a behavior are not able to be judged solely by the person emitting the behavior. Such judgments are the result of intersubjective processes which have shaped the meanings we attribute to behaviors. In this sense, meanings do not emerge *de novo*, they are jointly constructed. Thus, the attachment of meaning to experiences, via reflection, always requires a sense of that experience being potentially or actually, shared or experienced, by another (Stern, 2004). In this sense, metacognition can be seen as occurring not just in the mind of one person but intersubjectively. The ideas we form and reflect upon about ourselves and others require the real *or imagined* presence of other people who are thinking and relating or could think about and relate to the idea that we are forming of ourselves and others (Cortina and Liotti, 2010). Examples of real persons who might in the present think about an individual’s thoughts and experiences could include a partner, friend, competitor, or family member. Examples of persons who might be *imagined* to think about an individual’s thoughts and experiences could be a deceased parent, a child or grandchild not yet born, or a significant other who is not present at the moment, but who could be present in the future. Consistent with identity and social identity theories that stress that self-relating is, by definition, embedded in a social and interpersonal context (Stets and Burke, 2000) the present or imagined other can be thought of as someone to whom our ideas of ourselves and others are addressed (Hasson-Ohayon et al., 2017; Lysaker and Klion, 2017). From a slightly different angle, developmentally there are no isolated human thinkers in any ordinary sense. Thinking always emerged from inherited meanings, and meaning is inherited intersubjectively. To that degree, intersubjectivity is also a condition for the possibility of metacognition.

One potential reason why research has paid limited attention to the role of intersubjectivity in metacognition is that metacognition is often assessed in laboratory or analog settings where the meaning of the task is nominal or assumed not to vary significantly in terms of the meanings assigned to it. Many laboratory tasks are unlikely to be personally meaningful to the participant. For example, solving an abstract sorting task may have little personal relevance and so the role of meaning and influence of intersubjective experience may be negligible or virtually undetectable. Reflections about behavior in laboratory tasks are further constrained a highly circumscribed manner as either correct or incorrect. Hence, the meanings different persons might generate about the task would not be presumed to vary. For example, in these kinds of tasks, most people would agree on whether they made a correct response.

While the neglect of the relationship of intersubjectivity and metacognition is understandable given measurement approaches, a failure to consider the role of intersubjectivity limits a full understanding of the links between metacognition with functioning as well as approaches to addressing metacognitive deficits. In response to these limitations, in part, recent research on metacognition has taken a new direction which may allow the role of intersubjectivity to come into sharper focus. This work, which specifically focuses on disturbances in metacognition in schizophrenia, uses experimental tasks which ask individuals to recall and describe their experiences and response to psychosocial challenges. Concretely, the tasks in this emerging experimental paradigm require participants to provide a narrative, in which they form ideas about emotionally salient real-life experiences, to which meanings may be explicitly or implicitly assigned and then researchers can explore the extent to which complex metacognitive activities were evidenced (Lysaker et al., 2005).

To date, the majority of work using this method has had the aim of understanding co-occurring alterations in self-experience and the associated interpersonal challenges to relate to others in schizophrenia (Lysaker et al., 2019). In this work, conjoint disturbances in the ability to form a sense of how others perceive or value experience and an inability to see relationships of fragments of experience with one another, are thought to result in the loss of capacity for the experience of interiority, historicity, agency, and intimacy. The impetus for the need to understand metacognitive processes as they occur during reflections about challenging aspects of life includes longstanding observations that persons diagnosed with schizophrenia, and more broadly psychosis, experience difficulties in binding information into a larger sense of self and sustain meaningful connections with others (Bleuler, 1950; Freud, 1957), often finding intersubjectivity challenging (Fromm-Reichmann, 1954; Sullivan, 1962). It has also been inspired by more contemporary work reporting many persons diagnosed with schizophrenia might experience difficulties recognizing others’ emotions, motives, and personal qualities (Pinkham, 2014; Bell et al., 2017; Buck et al., 2018) while also showing relatively low sense of self-clarity (Hasson-Ohayon et al., 2014) and low ability to describe their own emotions (Fogley et al., 2014).

This approach to metacognition may thus offer new opportunities to reconsider concrete aspects of the relationship between metacognition and intersubjective processes because it is explicitly concerned with larger meanings people form (or do not form) along with the need to address psychological and social aspects of a person’s life in the world. To that end, this paper presents an emerging definition of metacognition as an integrative process and then discusses definitions of intersubjectivity. We will then discuss research on this methodology and findings on the relations of metacognitive deficits and the phenomenology of schizophrenia. With this foundation, we will explore three issues that this literature led us to consider: how variation in metacognitive capacity and intersubjectivity are related, how that relationship is closely tied to health, and finally how treatments that address metacognitive deficits in schizophrenia always have an underlying intersubjective element.

## Metacognition: An Integrative Model

Following Flavell (1979) original definition of metacognition as complex and multifaceted processes, as discussed above, a range of paradigms for measuring metacognition have been developed with broad applications to the fields of cognitive, educational, personality, and clinical psychology. In an effort to piece together research on metacognition and mental health in particular, an integrative model of metacognition has been proposed (Lysaker et al., 2020) which conceptualizes metacognition as a spectrum of activities which require the recognition and potential integration of thoughts, feelings and embodied experience. Regardless of whether recognizing, monitoring one’s abilities, or altering one’s strategies, this model suggests that metacognitive activities require the abilities to both notice basic and distinct emotional, cognitive, and embodied experiences and to understand the relationships they have to one another. In other words, metacognition allows for multi-sensory, cognitive, and behavioral elements to be integrated into a coherent and usable representation of experience.

For example, while driving, someone might notice that they are driving unusually quickly, their face is warm, they are frustrated with other drivers around them, and can think of nothing satisfying to eat later in the evening. In this situation, a person might then connect these individual things and realize he or she is upset. This person might then realize that being upset is specifically about a conflict with a family member. This realization can then be a subject for reflection and that experience of being upset might be connected to other experiences. Comparing and contrasting it to other times a person was upset might lead to a realization of a pattern of relating which can then be used to regulate a current state or arousal which could again be a subject for reflection and lead to further metacognitive knowledge.

Metacognition may, therefore, involve activities which vary in at least two ways. First, metacognitive activities may vary in terms of their concern with more discrete and limited aspects of experience which are considered on their own versus broader phenomena which are comprised of many elements with differing relationships with one another. Some metacognitive acts may involve the detection and reflection upon discrete and highly specific mental experiences (e.g., identifying specific thoughts, emotions, or wishes) others require the integration or synthesis of many discrete phenomena into something broader (e.g., synthesizing one’s own intentions, thoughts, and feelings into a sense of oneself as a unique being; Lysaker and Dimaggio, 2014; Lysaker and Hasson-Ohayon, 2014). Metacognition may second vary according to the subject of reflection. Reflection concerning oneself, for example, can be distinguished from reflection about others and the use of that knowledge (Semerari et al., 2003). The reasoning here is that each calls the assembling of different kinds of information and their translation into different actions.

While these different aspects of metacognition would be assumed to deeply influence one another, it is natural that they could be meaningfully measured using different paradigms. In the study of psychopathology, these have included, for example, assessments of the accuracy of particular judgments about memory and task performance, beliefs about one’s attitudes, awareness of the limits of one’s own knowledge, and the degree of integration of senses of self, others, and one’s place in one’s community (Semerari et al., 2003; Lysaker et al., 2013). These different aspects of metacognition would also naturally be multidetermined and supported by a range of social, psychological, and biological processes. Deficits in metacognitive capacity in the field of mental health have been linked, for example, to neurocognitive deficits, interpersonal trauma, insecurity of attachment, stigma, and social alienation, any of which could restrict the ability to form and share ideas about experience (Lysaker and Klion, 2017). A major point that is raised in this paper is that intersubjectivity is a factor intertwined with metacognition, suggesting that the sense of self and the other as subjective human being is essential for the application of metacognitive acts. This applies for metacognitve acts that are more discrete, as well as to more synthetic ones. Of note, applying metacognitive acts that are relatively discrete might require less engagement in intersubjectivity, although the genesis of meaning, even with regard to specific discrete event, involves social aspects.

## Intersubjectivity: Definitions

Like the construct of metacognition, the construct of intersubjectivity refers to a continuum of experiences that occur between people rather than solely in the mind of one person. In a broader sense, intersubjectivity refers to what is occurring between two minds (Beebe et al., 2005). In a narrower sense, it points to experience of shared states of mind (Trevarthen, 1998) or a mutual recognition and understanding of others’ subjective experiences (Benjamin, 1990; Stern, 2000). Cortina and Liotti (2010) have emphasized that intersubjectivity is a unique kind of human communication and is a precondition for human beings to describe experience and ultimately understand one another. Intersubjectivity is believed to develop in recognizable stages. For example, Trevarthen (1998) proposes that intersubjectivity is present in early infancy in a form he terms “primary intersubjectivity.” In this stage, the infant and caretaker communicate though the mutual and ongoing regulation of bodily rhythms and changing levels of affective arousal. Stern (2000) concretely proposes that a subjective sense of self tends to emerge from these encounters roughly around the age of 7–9 months and it is during this time that the infant begins to have a working sense, albeit pre-verbal, of their experiences and those of the caregiver.

With development, the capacity to symbolize experience is then proposed to develop next as the infant and caretaker communicate with each other in more complex ways in what is termed “secondary” as opposed to “primary intersubjectivity” (Trevarthen, 1998). This is believed to be facilitated by the development of the ability to shift attention from a world consisting of only two persons to one that could be seen by a third person which then allows for other ways for the infant and caretaker to understand one another (Tomasello et al., 2005). This then allows for further translation of experience into symbols to be shared with others leading to what Stern (2000) referred to as the verbal self; a self expressed in words and then later the narrative self, or the self as positioned with narratives or complex sequences of events.

Fernyhough (2008) thus proposes that these experiences of intersubjectivity support children to develop both basic and advanced forms of imagination, narration, and language abilities, leading to the enhanced motivation to share experience and apply more sophisticated reasoning regarding mental states of self and other. Consistent with Piaget (1995) work on the social origin of mental functions and thinking about continuing development, he utilizes Vygotsky (1997) conceptualization of the dialogic nature of the higher mental functions among children and emphasizes that the origin of reflectiveness is social and that any “individual” mental functioning is based on processes of interpersonal internalization (Fernyhough, 2008).

Importantly, as in the case of metacognition, the failure to develop the capacity for intersubjectivity could result from multiple sources at different points in time. For example, early in development this process could be challenged if either the caretaker or infant has no or limited sense of the others bodily rhythms or affective states. Gallese (2003) has suggested that human beings possess discernable and separate neurological systems which mirror the experience of one another, termed networks of mirror neurons, and that disturbances in those processes could disrupt the foundations for relatedness. Others have suggested that early relational trauma and disturbances in attachment also disrupt or damage these processes (Gumley and Liotti, 2019) resulting in a broad range of disturbances in affect regulation and mentalization (Fonagy, 1991). Social forces related to inequity and stigma and its internalization have also been suggested as barriers to having a sense of the legitimacy of one’s own subjectivity, compromising ultimately the potential for intersubjectivity (Bassman, 2000; Hasson-Ohayon, 2012).

## Investigations of the Role of Metacognitive Deficits Into the Phenomenology of Schizophrenia

Studying both phenomenology and psychosocial function in schizophrenia, research rooted in the integrated model of metacognition has explored how well persons can integrate information within a narrative to form an idea of the self, others, one’s place in the larger community and use that knowledge to respond to psychosocial challenges. The research paradigm begins by soliciting a spontaneous spoken narrative of the participant’s life and experience of psychosocial challenges. A rater then using the Metacognition Assessment Scale-Abbreviated (MAS-A; Lysaker et al., 2005; Lysaker and Klion, 2017) rates metacognition as it organically occurred in the telling of the narrative to the researcher. The material elicited is consequently relayed to someone who has explicitly asked about it, is of significant personal meaning, and is rife with meanings that are inextricably linked to how others might think about that material and how others might think about the meanings the participant assigns as he or she talk about it.

The MAS-A was inspired by an earlier scale, the Metacognition Assessment Scale (MAS; Semerari et al., 2003). It was developed in 2004 retaining several basic characteristics of the original MAS (Semerari et al., 2003). Whereas the MAS was designed to assess the ratio of the number of opportunities to occurrences for certain metacognitive acts within a psychotherapy session, the MAS-A instead quantitively assessed the overall capacity for metacognition. To do this, the MAS-A departed from the MAS and conceptualized metacognitive activities as divisible into a series of levels which range from elemental to more complex activities, with each gradation reflecting an increase in complexity and each more complex level requiring satisfactory function at the previous more elemental level. Concretely then the MAS-A generates a single score for each dimension (self-reflectivity, awareness of the other, decentration, and mastery) with higher scores reflecting metacognitive acts in which more complex integration had occurred. Thus, accuracy or correctness is not assessed. Scores instead reflect the degree to which material is integrated when participants talk about themselves, others, their communities, and the use of that knowledge to respond to distress.

Research using this paradigm has successfully detected poorer metacognitive abilities among adults diagnosed with schizophrenia as opposed to other conditions (e.g., Hasson-Ohayon et al., 2015) and revealed that having relatively greater levels of metacognitive deficits was linked to poorer prospective and future functioning and symptom severity (McLeod et al., 2014; Arnon-Ribenfeld et al., 2017; Lysaker et al., 2020). Most recently and pertinent to the reconsideration of the relationship of metacognition and intersubjectivity, poorer metacognition has been uniquely linked to the experience of impoverished social connections despite having no association with prosocial behavior (Fisher et al., 2020). It was also linked to reduction in empathy (Bonfils et al., 2019), psychological resources needed for social functioning independent of symptoms (Gagen et al., 2019), and prospective deficits in expressing emotions to other (Austin et al., 2019). It has lastly also been found to have a unique link to reduction in positive forms of self-compassion (Hochheiser et al., 2020). Of note, metacognitive functioning has also been found to be responsive to interpersonal interventions including psychotherapy (Lysaker et al., 2019).

## Reconsidering the Relationship of Metacognition and Intersubjectivity

Initially we suggested that one reason for the relative neglect of the relationship of metacognition and intersubjectivity was reliance on experimental paradigms in which the effects of intersubjectivity were relatively negligible. In response we have reviewed research which has used an emerging experimental paradigm require participants to provide a narrative, in which the thoughts and experiences of other should naturally affect the meanings participants explicitly or implicitly assign to experience. Reflecting on this work provides an opportunity to consider three issues:

1. Variations in metacognitive capacity and intersubjectivity mutually affect one another.

As illustrated in the clinical research reviewed above, persons diagnosed with schizophrenia with relatively greater metacognitive impairments have problems which could be seen as hallmarks of disturbances in intersubjectivity. They have fewer intimate relationships, greater difficulties recognizing the thoughts and feelings of others and greater difficulties empathizing with others (e.g., Hasson-Ohayon et al., 2015). Considered theoretically, at the simplest level, it is likely that these co-occurring deficits in metacognition and intersubjectivity mutually influence one another. For one, with a relatively fragmented and non-integrated sense of self, it would be more difficult to understand the experience of others and forge an empathic connection. As suggested by Dimaggio et al. (2008), to adequately grasp the experience of other people we have to be able to form an idea of ourselves experiencing circumstances similar to theirs, something likely to be impeded by low self-reflectivity. For example, without the ability to form an integrated sense of oneself when facing a particular dilemma, it is hard to imagine that one could jointly share another person’s experience of that dilemma. This is consistent with hypotheses about how mirror neurons support social function (Gallese, 2001) as well as empirical findings in schizophrenia research that better self-reflectivity predicts emotion recognition independently of theory of mind abilities (Lysaker et al., 2014). Considered concretely, losses in metacognitive function may also render previously coherent sense of social exchange confusing and senseless, resulting in a loss of agency and sense of who one is in the larger social world, perhaps similar to the way in which Kafka’s characters wake up one day and their position in life no longer make sense (Kafka, 1971). With reduced self-reflectivity, intersubjective exchanges may then become more threatening and something to be avoided. Social exchanges may be overwhelming when another person can form a more coherent and integrated sense of ourselves than we can, resulting in the experience that the other person can know us far better than we know ourselves. With limited metacognitive resources, social exchanges may stir up aspects of ourselves which are not integrated and accordingly which can be experienced as degrading an already limited sense of cohesion, again resulting in an understandable wish to avoid intersubjective connections (Lysaker and Lysaker, 2010).

From the opposite point of view, substantial alterations in the capacity of quality of intersubjective experience may also affect metacognitive function. If the ability to tolerate the presence of others in intimate ways and think with them, for example, as secondary to interpersonal trauma, one can readily imagine that the metacognitive capacities for self-reflectivity, awareness of others, as well as mastery and decentration might be challenged. Without people to think with, it seems natural that the sense persons have of themselves and others will become less complex and integrated. Without people to share ideas of oneself and others there would naturally seem to be less to say and then less to reflect upon. This would be consistent with developmental models described above (e.g., Stern, 2004), as well as with models of disturbances in attachment and mentalization models of borderline personality disorder which link interpersonal trauma to reductions in the quantity and quality of mentalization (e.g., Fonagy, 1991; Gumley and Liotti, 2019). It is also consistent with finding linking childhood trauma, which is known to affect the early quality of intersubjectivity, to metacognitive function in schizophrenia (Aydin et al., 2016) and with observations that early disturbances in attachment predate the onset of psychosis (Macbeth and Gumley, 2008).

2. Disturbances in metacognitive capacity and intersubjectivity have a mutual and synergistic negative impact on mental health.

A second opportunity that emerges from the studies reviewed above is the exploration of how substantial decrements in metacognitive capacity and the quality of intersubjective experience have a mutual and potentially deeply entangled influence on mental health. In other words, these two phenomena not only affect each other but may be deeply involved in compromises to mental health when they are substantially disturbed. This possibility may help explain the kinds of profound disturbance mental illness can have upon the individuals’ life trajectories and help to see disability as more than a reflection of symptoms and skills deficits. For example, considering psychosocial functioning with deficits in metacognition, the deeper meaning of persisting when facing a challenge at work or in a relationship may be less apparent. In parallel, with weaker intersubjective experience, people may also find themselves as feeling they can only face a challenge at work or in a relationship alone. Consequently, the motivation to persist and not withdraw from work or relationships may be reduced heightened the possibility of a gradual withdrawal from functioning in the community. This is consistent with finding suggesting that a certain level of metacognitive function is needed for the experience of intrinsic motivation (Luther et al., 2017).

From a different angle, losses in metacognitive capacity and in healthy intersubjective function might also make it more difficult for persons to reject stigma and find a way to form a healthier idea of oneself. For example, it may be difficult to counter stigma if one’s sense of self is fragmented and a connection to other who love and hold you in esteem is unavailable. This idea is in line with findings by Nabors et al. (2014) that better metacognitive capacity is related to greater ability to reject stigma among persons with schizophrenia, and with findings by Hasson-Ohayon et al. (2014) that low sense of self-clarity leaves one vulnerable to the internalization of stigma without alternative narrative that can emerge from intersubjective experiences.

Turning to the process of recovery from serious mental illness, from the view that for many diagnosed with schizophrenia, disability is intimately related to isolation and a diminished sense of self, and wellness to a return to a sense of connection to other and coherent sense of who is in the world (Leonhardt et al., 2017). Here again joint contribution from metacognitive disturbances and alterations in the quality of intersubjective experience seem likely. Both metacognitive capacity and intersubjective experience seem necessary for a sense of belonging to a larger group in which its members support and protect one another. They are also needed for a coherent sense of oneself as knowable by others and as potential subject of compassion.

Of note, it might be that having either high metacognitive abilities or high sense of intersubjectivity can serve as protection from possible negative implications of disturbances in each. For example, with healthy intersubjective connections, persons might be able to borrow the capacities of trusted other to help them form integrated ideas of the self or other. In parallel, with intact metacognitive capacities, persons might also be able to find ways to tolerate the distress that comes from the loss of healthy intersubjective function and find ways to reform connections with others. This is consistent with findings that having higher levels of metacognition ability of Mastery, the ability to use metacognitive knowledge to respond to psychosocial challenges, may neutralize the effects of poor attachment on symptoms of borderline personality disorder (Outcalt et al., 2016).

3. Addressing metacognitive disturbances require an intersubjective approach.

While considering the relationship between metacognition and intersubjectivity, a final point should be made with regard to psychosocial treatments that seek to address metacognition or health, in general and among persons with serious mental illness. Most directly, one possibility is that psychosocial treatments are likely to be more effective if and when they attend to intersubjective experience. If sense making and the integration of experience occurs largely between persons, and as metacognition and intersubjectivity are deeply related phenomena which influence one another and health, then enhancing intersubjectivity is likely to be a major path toward enhancing metacognition.

In concrete terms, this suggests that treatments need to consider people’s intersubjective experience of treatment itself and not just be conceptualized as a primarily educational activity. As suggested by the intersubjective metacognitive model of psychotherapy with psychosis outlined by Hasson-Ohayon et al. (2017), clients’ and therapists’ characteristics affect the therapeutic dialog. Accordingly, there is a need to consider differences in the personal narratives of the client and therapist with regard to their roles in the mental health system, the role of the mental health system itself, the meaning of mental health and lack of it, and the role of the client within psychotherapy.

Most commonly this might be thought of developing a shared understanding between two people, with both minds having their own ideas but sharing the experience of encountering each other (Buck et al., 2015). Such an understanding would be foundational then for the therapist and client to think about the patient and jointly begin to try and integrate information in ways that are meaningful for the patient. A point worth emphasizing is that the recapturing of metacognitive capacity cannot come from the clinician. It is not a skill imparted by someone else or a piece of wisdom or insight delivered from the outside. This idea is in line with Gerson’s (1996) psychoanalytic observation that the therapist is engaged in the process of knowing with the client, and does not decide by him or herself something about the client that should be provided to him or her. Thus, metacognition is not something the therapist teaches; rather it is a sense of the self as existing, as thinking, as interacting, and as evolving within an interaction.

Evidently, there are emerging treatments which make this idea explicit including mentalization-based treatment (Brent and Fonagy, 2014) and metacognitive-based integrative psychotherapies such as Metacognitive Reflection and Insight Therapy (MERIT, Lysaker and Klion, 2017). In both, one aim is to address alterations in the experience of the self and others via intersubjectivity (Ridenour et al., 2018). In mentalization-based treatment, persons develop the ability to form more secure attachments and then become able to develop the kinds of senses of self and others needed to find their way to a meaningful life. MERIT by contrast offers a set of eight key elements which should be enacted in a typical session to enhance metacognition. These elements are intersubjectively driven and include such interventions as the therapist sharing with the patients his or her thoughts or feelings that are relevant to what is taking place in the intersubjective space (Hasson-Ohayon et al., 2017).

Even if intersubjectivity is not considered explicitly in treatment, it may still be considered implicitly and understood as a key therapeutic mechanism. For example, Narrative Enhancement Cognitive Therapy (NECT, Yanos et al., 2011) seeks to reduce of self-stigma via the challenges of beliefs and the re-creation of self-narrative. By applying narrative techniques that are related to the capacity for reflection, this intervention may enhance metacognition. In addition, the effects of other more focused interventions (e.g., social cognition and interaction training by Roberts et al., 2015) may also be enhanced by assimilating an intersubjective metacognitive framework (Hasson-Ohayon, 2012). Similarly, metacognitive training exercises may be potentially conceptualized as affected by the intersubjective experiences this treatment allows leading to larger metacognitive gains (Moritz et al., 2018). Of note, as mentioned above, these phenomena may also affect one another in the opposite direction. For example, the development of metacognitive capacity may allow for the development of therapeutic alliance as suggested by Davis et al. (2011) which may then allow for the development of healthy intersubjective experience.

## Summary and Implications

We have suggested that the relationship between metacognition and intersubjectivity has been neglected, probably in large part due to paradigms of metacognition that are concerned with specific tasks which focused on accuracy and biases. To begin to reconsider the relationship between metacognition and intersubjectivity, we have turned to advances in conceptualization and measurement of metacognition specifically in the area of schizophrenia, a condition in which substantial disturbances in both phenomena have long been noted. Following this work, we have proposed at least two keys ways in which metacognition and intersubjectivity are related: they influence one another, and are jointly affect health. Concerning psychosocial treatment, we have suggested that recovery from metacognitive disturbances is not something that occurs just in the mind of one person after appropriate training. It is necessarily a matter of human connection and the establishment of mutual and shared understanding between persons. Our view is that there are dangers of disembodied training which on its own may only reinforce alienation and ultimately be a barrier to the kinds of complex metacognitive acts which are required for persons to find a way to manage complex mental health needs and find a way to a fully satisfying life.

With this message in mind, a few points should be raised for future work. First, while evidence is accumulating about the relationship of metacognition and recovery from schizophrenia, the role of intersubjectivity in the recovery process has been less studied. While we have explored literature on substantial alterations in metacognition, it is unclear to what extent intersubjective experience plays a role in persons who experience more negligible alterations. In addition, while we have advocated intersubjective consideration into treatment of persons with schizophrenia, this is yet to be fully validated empirically. A recent study showed that the use of an intersubjective intervention by the therapist, improved outcome for persons with schizophrenia (Lavi-Rotenberg et al., in press). Notably, additional studies are needed to support the joint effect of metacognition and intersubjectivity on health and therapy outcome. It is also unknown whether other treatments that were not discussed here and may address more cognitive component or focus on intersubjectivity solely might be equally or as effective.

Future research is needed which employs a broad range of experimental paradigms for measuring metacognition and health among persons with and without disabling mental conditions. Careful longitudinal designs which take intersubjective experience into account could allow for the development of more nuanced accounts of the relationships of different aspects of metacognition and intersubjectivity with one another and their linkages with human adaptation.

## Author Contributions

IH-O and PL conceptualized the theoretical statement and main messages. HM and AG contributed to writing and presentation of ideas.

## Conflict of Interest

The authors declare that the research was conducted in the absence of any commercial or financial relationships that could be construed as a potential conflict of interest.
